# Supplementary material for: Profiles of social isolation and loneliness as moderators of the longitudinal association between uncorrected hearing impairment and cognitive aging
Source: Commun Psychol. 2025 Jul 9;3:101. doi: 10.1038/s44271-025-00277-8 (PMC12240851; doi:10.1038/s44271-025-00277-8)
Supplement: Supplementary file 2 — Supplementary material [file 44271_2025_277_MOESM2_ESM.pdf]

Supplementary Table 1. Comparisons of fit indices for the models with immediate recall as outcome variable.

	Number of parameters	AIC	BIC	-2 log likelihood	Deviance	χ^2	Df	p
M1: Empty model	3	496412	496441	-248203	496406			
M2: With retest effects	10	495640	495738	-247810	495620	785.687	7	***
M3: With covariates	16	481937	482094	-240953	481905	13714.947	6	***
M4: Adding hearing impairment mean	17	481552	481719	-240759	481518	387.503	1	***
M5: Adding hearing impairment linear change	18	481448	481625	-240706	481412	105.173	1	***
M6: Adding hearing impairment quadratic change	19	481392	481579	-240677	481354	58.455	1	***
M7: Adding social isolation/loneliness profiles	22	481145	481361	-240551	481101	252.745	3	***
M8: Adding **random effects** for the **linear change** in hearing impairment	24	480906	481142	-240429	480858	243.038	2	***
M9: Adding **random effects** for the **quadratic change** in hearing impairment	27	480900	481165	-240423	480846	12.152	3	**
**M10: (final, most parsimonious model)**: Adding **interaction** between the **linear change** hearing impairment and the social isolation/loneliness profiles	30	480876	481171	-240408	480816	29.822	3	***

Notes. To compare the fit all models were refitted with Maximum Likelihood (ML) instead of Restricted Maximum Likelihood (REML). ***p < .001; **p < .01; *p < .05.

Supplementary Table 2. Comparisons of fit indices for the models with delayed recall as outcome variable.

	Number of parameters	AIC	BIC	-2 log likelihood	Deviance	χ^2	Df	p
M1: Empty model	3	546326	546356	-273160	546320			
M2: With retest effects	10	545565	545664	-272773	545545	774.665	7	***
M3: With covariates	16	532958	533115	-266463	532926	12619.634	6	***
M4: Adding hearing impairment mean	17	532715	532882	-266341	532681	244.435	1	***
M5: Adding hearing impairment linear change	18	532618	532795	-266291	532582	99.416	1	***
M6: Adding hearing impairment quadratic change	19	532575	532761	-266268	532537	45.383	1	***
M7: Adding social isolation/loneliness profiles	22	532331	532547	-266143	532287	249.931	3	***
M8: Adding **random effects** for the **linear change** in hearing impairment	24	532071	532307	-266012	532023	263.319	2	***
M9: Adding **random effects** for the **quadratic change** in hearing impairment	27	532049	532314	-265998	531995	28.215	3	***
**M10: (final, most parsimonious model)**: Adding **interaction** between the **linear change** hearing impairment and the social isolation/loneliness profiles	30	531933	532228	-265937	531873	121.841	3	***

Notes. To compare the fit all models were refitted with Maximum Likelihood (ML) instead of Restricted Maximum Likelihood (REML). ***p < .001

Supplementary Table 3. Comparisons of fit indices for the models with verbal fluency as outcome variable.

	Number of parameters	AIC	BIC	-2 log likelihood	Deviance	χ^2	Df	p
M1: Empty model	3	884588	884618	-442291	884582			
M2: With retest effects	10	884167	884266	-442074	884147	435.142	7	***
M3: With covariates	16	875142	875299	-437555	875110	9037.208	6	***
M4: Adding hearing impairment mean	17	874993	875160	-437480	874959	150.751	1	***
M5: Adding hearing impairment linear change	18	874868	875045	-437416	874832	127.425	1	***
M6: Adding hearing impairment quadratic change	19	874814	875001	-437388	874776	56.084	1	***
M7: Adding social isolation/loneliness profiles	22	873790	874007	-436873	873746	1029.404	3	***
M8: Adding **random effects** for the **linear change** in hearing impairment	24	873588	873824	-436770	873540	205.944	2	***
M9 (**Final, most parsimonious model**): Adding **random effects** for the **quadratic change** in hearing impairment	27	873558	873823	-436752	873504	36.545	3	***

Notes. To compare the fit all models were refitted with Maximum Likelihood (ML) instead of Restricted Maximum Likelihood (REML). ***p < .001.

Supplementary Table 4. Correlations of study variables at the first measurement point with confidence intervals

Variable	1	2	3	4	5	6	7	8	9	10	11
1. Age											
2. Sex	-.01***<br>[-.02, -.01]										
3. Education	-.19***<br>[-.19, -.18]	-.08***<br>[-.08, -.07]									
4. Chronic conditions	.29***<br>[.29, .30]	.07***<br>[.05, .06]	-.15***<br>[-.15, -.14]								
5. Hearing impairment	.23***<br>[.22, .23]	-.11***<br>[-.09, -.08]	-.14***<br>[-.15, -.14]	.18***<br>[.17, .18]							
6. Non-isolated and less lonely	-.09***<br>[-.10, -.08]	-.09***<br>[-.09, -.08]	.12***<br>[.11, .12]	-.14***<br>[-.15, -.14]	-.08***<br>[-.08, -.07]						
7. Non-isolated and lonelier	.08***<br>[.08, .09]	.09***<br>[.09, .10]	-.11***<br>[-.11, -.10]	.14***<br>[.13, .14]	.07***<br>[.07, .08]	-.95***<br>[-.95, -.95]					
8. Isolated and less lonely	.004<br>[-.00, .01]	-.03***<br>[-.03, -.02]	-.01***<br>[-.02, -.01]	-.01***<br>[-.02, -.01]	.01***<br>[.00, .01]	-.11***<br>[-.12, -.11]	-.08***<br>[-.09, -.08]				
9. Isolated and lonelier	.03***<br>[.03, .04]	-.002<br>[-.01, .00]	-.03***<br>[-.03, -.02]	.02***<br>[.02, .03]	.02***<br>[.01, .02]	-.14***<br>[-.14, -.13]	-.10***<br>[-.11, -.09]	-.01***<br>[-.02, -.01]			
10. Immediate recall	-.32***<br>[-.32, -.31]	.10***<br>[.07, .08]	.35***<br>[.34, .35]	-.16***<br>[-.16, -.15]	-.18***<br>[-.18, -.17]	.11***<br>[.11, .12]	-.10***<br>[-.11, -.10]	-.02***<br>[-.02, -.01]	-.04***<br>[-.04, -.03]		
11. Delayed recall	-.31***<br>[-.31, -.30]	.11***<br>[.08, .09]	.34***<br>[.33, .34]	-.16***<br>[-.17, -.15]	-.17***<br>[-.17, -.16]	.12***<br>[.11, .12]	-.10***<br>[-.11, -.10]	-.02***<br>[-.02, -.01]	-.04***<br>[-.04, -.03]	.73***<br>[.72, .73]	
12. Verbal fluency	-.25***<br>[-.25, -.24]	.001<br>[-.00, .01]	.37***<br>[.36, .37]	-.12***<br>[-.13, -.12]	-.15***<br>[-.15, -.14]	.14***<br>[.14, .15]	-.14***<br>[-.14, -.13]	-.002<br>[-.01, .00]	-.03***<br>[-.04, -.03]	.47***<br>[.46, .47]	.46***<br>[.45, .46]

Note. Values in square brackets indicate the 95% confidence interval for each correlation. * $p < .05$. *** $p < .001$.

Supplementary Table 5. Multivariate multilevel model with fixed and random effects (verbal fluency as reference category)

Predictors	B (β)	SE	T(df)	CI	p
Fixed Effects					
(Intercept)	1.12 (0.06)	0.03	36.29(35130)	1.06 – 1.18	<0.001
Retest effects					
Wave 2	0.07 (.02)	0.01	11.31(342700)	0.06 – 0.08	<0.001
Wave 4	0.12 (.03)	0.01	20.38(338200)	0.11 – 0.13	<0.001
Wave 5	0.13 (.04)	0.01	25.58(331700)	0.12 – 0.14	<0.001
Wave 6	0.13 (.05)	0.01	25.30(319600)	0.12 – 0.14	<0.001
Wave 7	0.12 (.03)	0.01	15.20(352000)	0.11 – 0.14	<0.001
Wave 8	0.11 (.04)	0.01	13.88(317400)	0.10 – 0.13	<0.001
Wave 9	0.12 (.04)	0.01	12.25(311800)	0.10 – 0.13	<0.001
Between-subjects' effects					
Age (M)	-0.03 (-.21)	0.0004	-64.46(34230)	-0.03 – -0.02	<0.001
Sex (Female respondents)	0.15 (.07)	0.01	22.58(33170)	0.13 – 0.16	<0.001
Education	0.20 (.28)	0.02	85.45(33200)	0.19 – 0.20	<0.001
Chronic conditions (M)	-0.02 (-.03)	0.003	-8.78(33840)	-0.03 – -0.02	<0.001
Hearing impairment (M)	-0.08 (-.06)	0.004	-18.35(33760)	-0.09 – -0.07	<0.001
Cognitive domains					
Immediate recall	-0.02 (-.02)	0.004	–6.82(343700)	-0.03 – -0.02	<0.001
Delayed recall	-0.02 (-.02)	0.004	–6.64(343700)	-0.03 – -0.02	<0.001
Profiles of social isolation and loneliness (ref: non-isolated and low loneliness)					
Non-isolated and high loneliness	-0.18 (-.18)	0.01	-24.36(50540)	-0.19 – -0.16	<0.001
Isolated and low loneliness	-0.02 (-.02)	0.04	-0.53(53820)	-0.09 – 0.05	0.599
Isolated and high loneliness	-0.22 (-.22)	0.03	-7.53(53240)	-0.28 – -0.16	<0.001
Within-subjects' effects					
Age (C)	-0.02 (-.07)	0.001	-23.98(312600)	-0.02 – -0.02	<0.001
Chronic conditions (C)	-0.002 (-.002)	0.001	-1.55(286200)	-0.00 – 0.00	0.120
Hearing impairment (C)	-0.02 (-.02)	0.01	-5.19(73700)	-0.03 – -0.02	<0.001
Hearing impairment (C^2)	-0.03 (-.02)	0.003	-8.66(6073)	-0.03 – -0.02	<0.001
Interaction effects					
Immediate recall* Non-isolated and high loneliness	0.07 (.07)	0.01	11.81(343700)	0.05 – 0.08	<0.001
Delayed recall * Non-isolated and high loneliness	0.06 (.06)	0.01	11.43(343800)	0.05 – 0.07	<0.001

Immediate recall* Isolated and low loneliness	-0.12 (-.12)	0.03	-4.15(343700)	-0.17 – -0.06	<0.001
Delayed recall * Isolated and low loneliness	-0.11 (-.11)	0.03	-3.97(343700)	-0.17 – -0.06	<0.001
Immediate recall* Isolated and high loneliness	-0.02 (-.02)	0.2	-0.92(343800)	-0.07 – 0.02	0.359
Delayed recall * Isolated and high loneliness	-0.02 (-.02)	0.02	-1.00(343800)	-0.07 – 0.02	0.316
Immediate recall* Hearing impairment (C)	0.002 (.001)	0.01	0.27(344000)	-0.01 – 0.01	0.784
Delayed recall * Hearing impairment (C)	0.002 (.001)	0.01	0.27(344100)	-0.01 – 0.01	0.791
Non-isolated and high loneliness * Hearing impairment (C)	-0.01 (-.01)	0.01	-1.31(71520)	-0.02 – 0.00	0.189
Isolated and low loneliness * Hearing impairment (C)	-0.04 (-.03)	0.04	-1.16(70640)	-0.11 – 0.03	0.245
Isolated and high loneliness * Hearing impairment (C)	-0.03 (-.02)	0.03	-1.06(72910)	-0.09 – 0.03	0.288
Immediate recall* Non-isolated and high loneliness * Hearing impairment (C)	-0.03 (-.02)	0.01	-2.96(344100)	-0.04 – -0.01	0.003
Delayed recall * Non-isolated and high loneliness * Hearing impairment (C)	-0.01 (-.01)	0.01	-1.00(344200)	-0.03 – 0.01	0.319
Immediate recall* Isolated and low loneliness * Hearing impairment (C)	0.04 (.02)	0.04	0.89(343600)	-0.05 – 0.13	0.372
Delayed recall * Isolated and low loneliness * Hearing impairment (C)	0.03 (.02)	0.04	0.79(343600)	-0.05 – 0.12	0.430
Immediate recall* Isolated and high loneliness * Hearing impairment (C)	0.02 (.01)	0.04	0.45(343900)	-0.05 – 0.09	0.643
Delayed recall * Isolated and high loneliness * Hearing impairment (C)	0.001 (.01)	0.04	0.04(343800)	-0.07 – 0.07	0.969

	Estimates	SD	CI
Random Effects			
Residual variance	0.50	0.71	0.71 – 0.71
Intercept (variance)	0.29	0.54	0.54 – 0.56
Hearing Impairment (C) slope (variance)	0.04	0.21	0.20– 0.22
Hearing Impairment (C^2) slope (variance)	0.02	0.14	0.13 – 0.15
Intercept*Hearing impairment (C) slope (covariance)	0.08	-	0.05 – 0.11
Intercept*Hearing impairment (C^2) slope (covariance)	-0.25	-	-.29 – -0.21
Hearing impairment (C) slope* Hearing impairment (C^2) slope (covariance)	0.04	-	-0.03 – 0.11
ICC		0.38	
N		33731	
Observations		411075	
Marginal R^2 / Conditional R^2		0.194 / 0.499	

Notes. M = person-mean variable (between-subjects differences); C = person-mean centred variable indicating the linear change; C^2 = person-mean centred variable indicating the quadratic change; SD = standard deviation; CI = 95% confidence intervals; Marginal R^2 = variance explained by fixed effects only; Conditional R^2 = variance explained by fixed and random effects. The CIs in the random effects' variances correspond to their Standard Deviations, while in the random effects'covariances they correspond to the actual estimates.

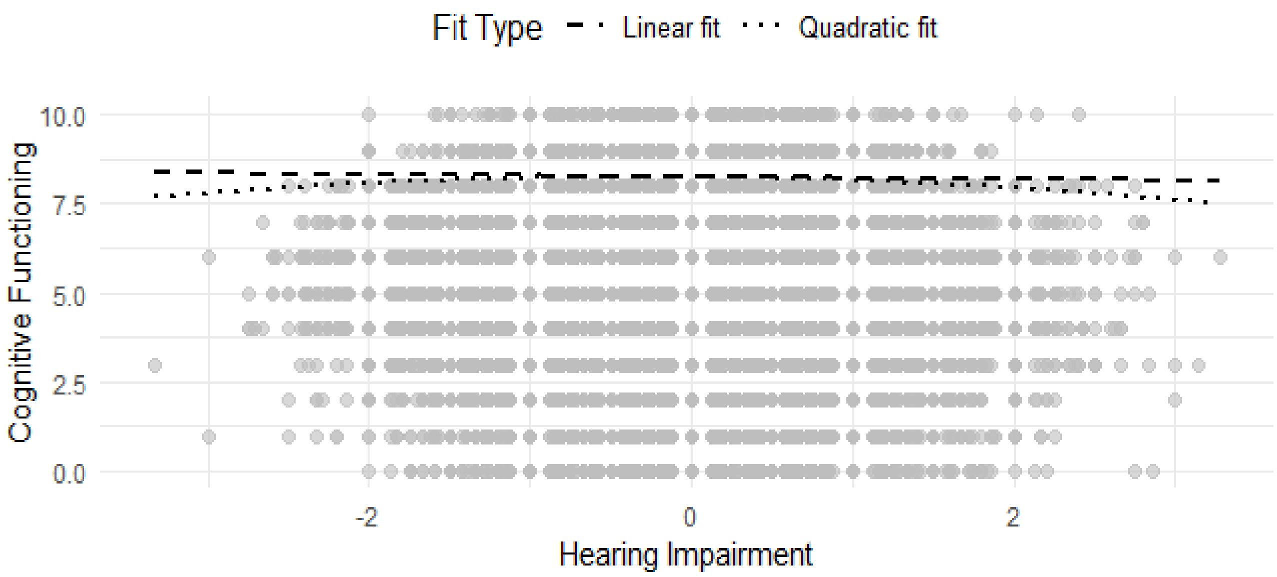

Supplementary Figure 1. Predicted cognitive functioning scores (immediate recall) by linear and quadratic change of hearing impairment (person-mean centred)

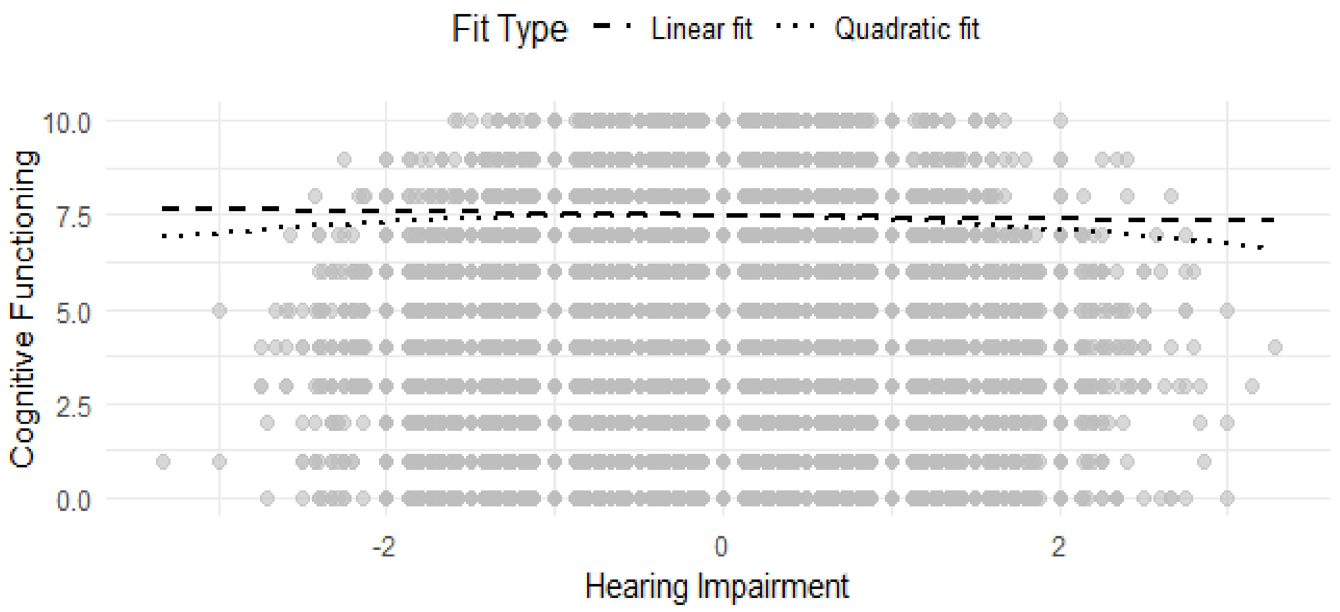

Supplementary Figure 2. Predicted cognitive functioning scores (delayed recall) by linear and quadratic change of hearing impairment (person-mean centred)

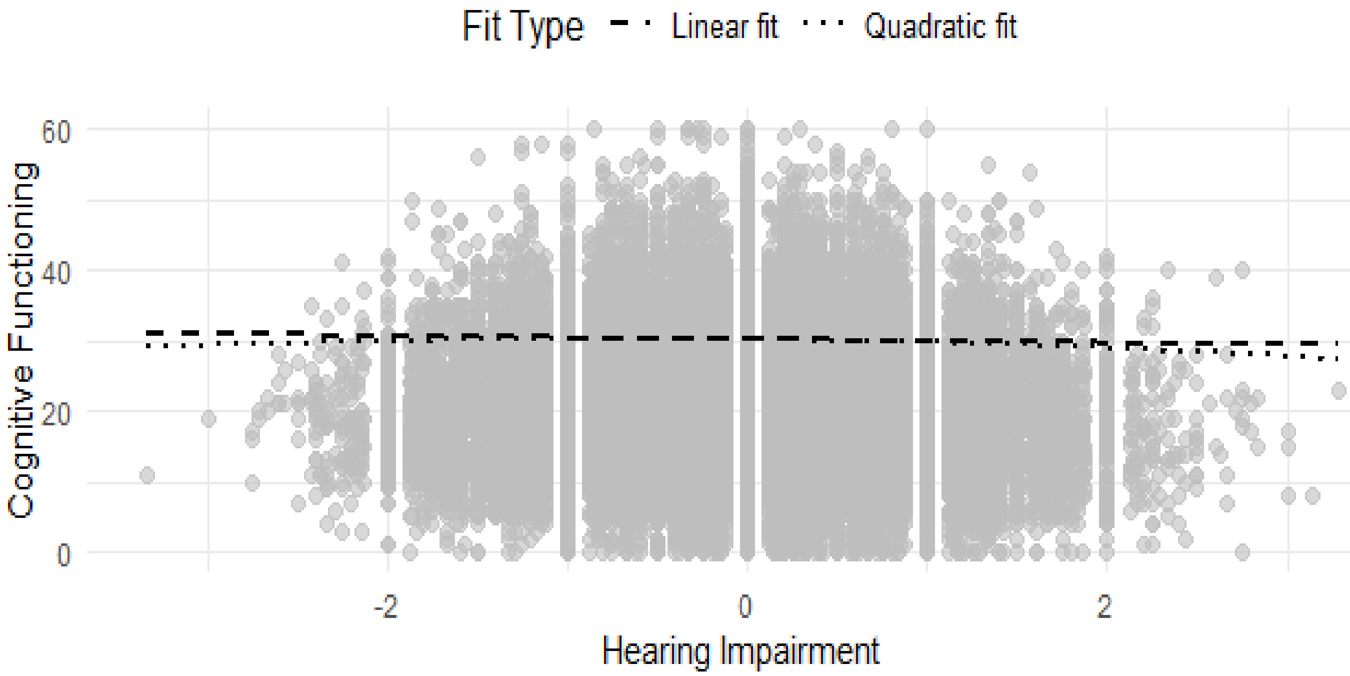

Supplementary Figure 3. Predicted cognitive functioning scores (verbal fluency) by linear and quadratic change of hearing impairment (person-mean centred)
